# Signaling by LncRNAs: Structure, Cellular Homeostasis, and Disease Pathology

**DOI:** 10.3390/cells11162517

**Published:** 2022-08-13

**Authors:** Revathy Nadhan, Ciro Isidoro, Yong Sang Song, Danny N. Dhanasekaran

**Affiliations:** 1 Stephenson Cancer Center, The University of Oklahoma Health Sciences Center, Oklahoma City, OK 73104, USA; 2 Laboratory of Molecular Pathology and NanoBioImaging, Department of Health Sciences, Università del Piemonte Orientale, Via Solaroli 17, 28100 Novara, Italy; 3 Department of Obstetrics and Gynecology, Cancer Research Institute, College of Medicine, Seoul National University, Seoul 151-921, Korea; 4 Department of Cell Biology, The University of Oklahoma Health Sciences Center, Oklahoma City, OK 73104, USA

**Keywords:** lncRNA, miRNA, epigenetics, cardiovascular disease, diabetes, cancer, Alzheimer’s, Parkinson’s, neurology, tumorigenesis

## Abstract

The cellular signaling network involves co-ordinated regulation of numerous signaling molecules that aid the maintenance of cellular as well as organismal homeostasis. Aberrant signaling plays a major role in the pathophysiology of many diseases. Recent studies have unraveled the superfamily of long non-coding RNAs (lncRNAs) as critical signaling nodes in diverse signaling networks. Defective signaling by lncRNAs is emerging as a causative factor underlying the pathophysiology of many diseases. LncRNAs have been shown to be involved in the multiplexed regulation of diverse pathways through both genetic and epigenetic mechanisms. They can serve as decoys, guides, scaffolds, and effector molecules to regulate cell signaling. In comparison with the other classes of RNAs, lncRNAs possess unique structural modifications that contribute to their diversity in modes of action within the nucleus and cytoplasm. In this review, we summarize the structure and function of lncRNAs as well as their vivid mechanisms of action. Further, we provide insights into the role of lncRNAs in the pathogenesis of four major disease paradigms, namely cardiovascular diseases, neurological disorders, cancers, and the metabolic disease, diabetes mellitus. This review serves as a succinct treatise that could open windows to investigate the role of lncRNAs as novel therapeutic targets.

## 1. Introduction

The genetic flow of information from DNA to protein via RNA has been held as the central dogma of molecular biology [1,2]. While this fundamental tenet of molecular biology is shaken to a certain extent by the discovery of reverse transcriptases that reverse the flow of information from RNA to DNA [3,4], proteins have remained as the critical downstream molecules that execute and regulate diverse cellular functions. The primary function of RNA has long been considered as the transcriber of genetic information from DNA and the template upon which the proteins can be built in the ribosomes [1]. However, the advent of transcriptomics and next generation sequencing technologies have unraveled the functional significance of several newer species of non-coding RNAs, that were once considered transcriptional noises or the dark matter of the genome. They include a family of microRNAs (miRNAs), circular RNAs (circRNAs), piwi-interacting-RNAs (piRNAs), small nucleolar RNAs (snoRNAs), and long non-coding RNAs (lncRNAs). LncRNAs are the non-coding RNAs that are greater than 200 nucleotides in length. In general, lncRNAs lack open reading frames and are not generally translated into proteins with a few exceptions of micropeptide-encoding lncRNAs [5]. To date, he GENCODE v35 has annotated 48,684 lncRNA transcripts and 17,957 lncRNA genes, while the NONCODE v5 has annotated the existence of 172,216 lncRNA transcripts and 96,308 lncRNA genes [6,7]. LncRNAs have been classified as sense-, antisense-, bidirectional-, intronic-, and intergenic-lncRNAs based on their genomic locations [8,9,10]. Sense lncRNAs are the ones whose sequence overlaps with the sense-strand of the protein coding sequence of a gene whereas antisense-lncRNAs are the ones whose sequence overlaps with the opposite antisense strand of a protein coding gene. Bidirectional lncRNAs are defined by the lncRNAs whose transcription is initiated by the same promoter region as that of the protein coding gene, but in the opposite direction. Intronic lncRNAs are the ones that are derived from the intron of another transcript. Long intergenic non-coding RNAs or the lincRNAs are those lncRNAs whose genomic loci do not overlap with any of the coding regions of the genome. However, in this review we have grouped all the sub-classes of lncRNAs as ‘lncRNAs’. Recent studies have documented a wide variety of mechanisms by which the lncRNAs regulate cellular homeostasis through their active as well as modulatory roles in diverse cell signaling pathways. In line with their critical roles, aberrant expression of lncRNAs have been shown to be associated with the pathogenesis and progression of several diseases.

Signaling by lncRNAs has the potential to interface with multitudinous factors of the cell signaling network due to their diverse functional roles such as serving as decoys, guides, scaffolds, and effector molecules. Recent progress in lncRNA research has unraveled the dynamics of lncRNA-mediated mechanisms in cellular homeostasis as well as pathogenesis. Analysis of lncRNA- interactomes that encompass proteins, RNAs, and lipids have pointed to the potential of lncRNAs as therapeutic targets. In this review, we discuss the structural and functional aspects of lncRNAs and emphasize the role of lncRNA signaling in human disease pathogenesis, focusing on four major disease paradigms, namely the cardiovascular diseases, neurological disorders, cancers, and diabetes.

## 2. Structural Features of lncRNAs

**Figure 1:**
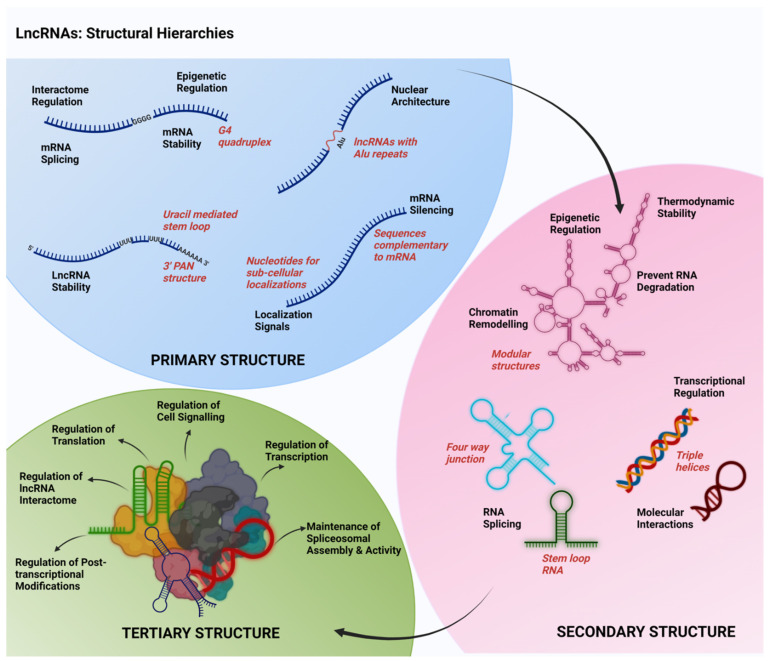
Structural hierarchies of lncRNA. The figure depicts the various levels of structural organizations of lncRNAs inclusive of their specialized modifications that provide them with their functional advantages. The primary structure of lncRNAs involve numerous specialized sequence modifications such as the presence of G4 quadruplexes, 3′ poly-A motifs, specific localization signals as well as presence of Alu retrotransposons that aid them in carrying out focussed functions. The secondary structure involves the modified primary sequences that attain improved structural hierarchies such as modules/domains, stem loops, triple helices, or even circular structures. Furthermore, the wide interactome of lncRNAs help them in bringing out broader levels of functions such as regulation of cell signaling, transcription, post-transcriptional modifications, and translation as well as maintenance of spliceosome assembly and activity.

To explore the multifarious role of lncRNAs as signaling molecules that can regulate diverse cardinal signaling cascades, a deeper understanding of the structural hierarchies of lncRNAs is very much required. Results from several studies have indicated that the structure of lncRNAs define their functional interactions with other macromolecules including DNA, RNA, and proteins. It appears that the primary and secondary as well as tertiary structures of lncRNAs play context-specific roles in the structural and functional organization of lncRNAs. Possible diversities in their primary, secondary, and tertiary structural conformations of lncRNAs that relate to their functional roles are discussed here (Figure 1).

### 2.1. Primary Structure

The primary structures of the lncRNAs are defined by their linear nucleotide sequences. Across the species, lncRNAs do not exhibit sequence conservation in their primary sequences other than with their promoter regions [11]. In general, lncRNAs are transcribed by RNA polymerase II from either the intergenic or the exonic regions or even from the distal protein coding regions of the genome. Certain lncRNAs are also transcribed by RNA polymerase III [12]. Once they are transcribed, they undergo post-transcriptional modifications, similar to the messenger RNAs (mRNAs), such as 5′ capping, 3′ poly-A tailing, and alternate splicing [13,14,15]. However, a few of the lncRNAs show specific primary sequence modifications that can be associated with their potential secondary structural motifs. One such specialized sequence is the presence of poly-adenylated nuclear (PAN) regions at the 3′ end of the lncRNAs MALAT1 and NEAT1 [16,17]. Unlike the classic poly-A tails, the poly-A rich region of MALAT1 and NEAT1 is preceded by two Uracil-rich motifs separated by a set of nucleotides, thus capable of forming a stem loop structure. These stem loop structures protect the poly-A coded region at 3′ end from deadenylation and associated degradation, conferring an enhanced stability to these lncRNAs [16,17]. Another specialized primary sequence that has been observed in a few of the lncRNAs (such as lincRNA-p21) is the presence of the transposable elements [18]. The most commonly observed transposable element in the lncRNAs is Alu retrotransposon, belonging to the family of short interspersed nuclear elements [15,18]. The lincRNA-p21 has been reported to possess two inverted repeats of Alu elements contributing to their helical structures. Mutation in these repeat elements has been shown to prevent the co-localization of lincRNA-p21 with NEAT1 in the nuclear paraspeckle structures [18].

Specific sequences such as the presence of Guanine-quadruplexes (G4) has also been reported to be associated with certain lncRNAs such as NEAT1, GSEC, and REG1CP [19]. The presence of G4 has been reported to affect the translation of mRNAs such as that of VEGF (vascular endothelial growth factor) and of TGFβ2 (transforming growth factor β2). This could be either through the modulation of the splicing or stability of mRNAs [19]. G4 also contributes to the association with polycomb repressive complexes (PRCs) and subsequent chromatin modifications (as shown by the lncRNA HOTAIR) through in vitro transcription and oligomer based experiments [20]. The lncRNA TERRA, which possess G-rich telomeric repeat sequences, has been identified with the G4 [21]. The presence of G4 enables TERRA to pair with Cytosine-residues in the DNA and form RNA-DNA duplexes resulting in R-loop structures. These R-loop structures help in the regulation of the telomere lengths in the differentiated cells [22].

Several other primary structure specific sequences can be attributed to the RNA- and DNA- interacting functions of lncRNAs. Primary sequences of the lncRNAs complementary to the miRNAs are attributed to the competitive endogenous RNA (ceRNA) function of lncRNAs that help in sequestration and inhibition of miRNAs [23]. There are nuclear localization sequences in lncRNAs such as PVT1, MALAT1, and BORG that facilitate their nuclear localization and associated functions [24]. Certain lncRNAs (e.g., MALAT1) possess sequences complementarity to the recognition motifs in U1 snRNPs (small nuclear ribonucleoproteins), which aid their binding and recruitment to the chromatin regions to facilitate pre-mRNA splicing [25]. Thus, the primary sequence of the lncRNAs provides clues to their predicted secondary structural modifications as well as sequence-dependent interactions with other RNAs and proteins.

### 2.2. Secondary Structure

Since lncRNAs exhibit a low sequence conservation across various species, the specificity of their interactome and functional diversity are rather dependent on their secondary and tertiary structures [26]. LncRNAs possess numerous secondary structural modifications that enhance their thermodynamic stability. These include bulges, junctions, hairpin loops, stem loops, internal loops, helices, subdomains, and pseudoknots, most of which involves the non-Watson Crick base pairing [27]. In some cases, the formation of secondary structure involves ribose backbone interactions [27,28,29]. These secondary structures determine their functional interactions with proteins, DNAs, and other RNAs [30]. Numerous studies have shown that several lncRNAs have modular structures that are comprised of ten to a hundred nucleotides in length. LncRNAs such as SRA, MEG3, and HOTAIR possess such modules in their secondary structure. These modules are not just independent folding units, but serve as independent functional units as well. For instance, HOTAIR possess four modules, namely D1-D4, wherein the 5′ D1 module interacts with PRC2, while the 3′ D4 module interacts with lysine-specific demethylase 1 (LSD1) to carry out their chromatin associated epigenetic regulatory functions [30,31]. HOTAIR, which has four independent folding modules/domains, possesses 56 helical regions, 19 junctions, 34 internal loops, and 38 terminal loops [30]. The multi-way junctions that are formed at the junctures of the stem loop structures are also significant as they define numerous tertiary structures as well as interactions of the lncRNAs. For instance, in human MEG3, the highly conserved H10 and H11 stems are oriented by the three-way junction J3 in their D2 module [31]. These junctions confer rigidity to the lncRNA secondary structures. The internal loops modulate the flexibility of the helical stems, which would in turn enhance numerous intramolecular as well as intermolecular interactions of the lncRNAs. The asymmetric right hand turns (RHTs) are examples of recurrent internal loops which possess specific 3′ and 5′ single stranded nucleotide stretches that interacts non-canonically to form the functional units. In U6 snRNP, such RHTs serve as the receptor for pre-messenger RNA splicing protein [32].

One of the prominent secondary structures of lncRNAs is the triple helix structure. It is a clover-leaf or four-way junction structure located at the 3′end, which protects the lncRNAs from degradation. During normal instances, post transcriptional processing of lncRNA involves the cleavage and polyadenylation specificity factor 73 (CPSF73)-mediated endonucleolytic cleavage and subsequent polyadenylation, which is essential for the RNA stability. However, a triple helix is formed when the cleavage is induced by Ribonuclease P, instead of CPSF73, thereby removing a conserved tRNA structure from the 3′ end. Further processing is carried out by Ribonuclease Z that generates a short adenine rich strand, resulting in a UAA triple helix at the 3′ end. LncRNAs such as MALAT1 and NEAT1 exhibit a similar triple helical structure at their 3′ end, which enhances their thermodynamic stability [16,17,33,34,35]. Another secondary structure is the double stem loop structure, which is mostly associated with the lncRNAs involved in chromatin remodeling. Such lncRNAs act in *trans* by interacting with the chromatin modifying enzymes. GAS5 and HOTAIR are examples of lncRNAs that possess a double stem loop structure to carry out chromatin remodeling [36,37,38]. For instance, GAS5 possesses A-form double helical structure that interacts with DNA binding domain of steroid receptors in a sequence specific manner to repress the steroid-mediated transcriptional mechanisms [39].

It should be noted here that the predicted secondary structures of many of the lncRNAs are based on in vito studies. They are derived from structural modeling using computational methods or biophysical, chemical, and enzymatic probing using purified lncRNAs [15,40]. The limitations of these analyses are that they do not take into account the endogenous interacting partners of lncRNAs that nucleate and/or stabilize the secondary structures of the lncRNAs in vivo. However, the complementary approaches using structural modeling and enzymatinc foot printing have to a great extent provided clues to the functional role of secondary structures [40].

### 2.3. Tertiary Structure

In addition to the secondary structure, the tertiary structure also attributes to their diverse interactome, which enables them to carry out their varied and dynamic functions. Major structural features that contribute to the tertiary structures of lncRNAs include T-loops, kink and hook turns, tetraloop receptor interactions, ribose zippers, minor groove triples and A-minor interactions, pseudoknots and kissing loops, G-quadruplexes, and/or triple helical modifications [41]. Though recent findings from several laboratories are unfolding the diverse tertiary structures in lncRNAs and their association with various functions, the current knowledge on these is limited. One of the lncRNAs whose 3-dimensional structure has been well explored is MEG3. A conserved hairpin H11 in D2 module/domain and the region H25-H27 within the D3 module/domain are the prominent RNA motifs necessary for p53 stimulation. Further, six of the repeated sequences in H27 are complementary to the sequences in the terminal loop H11 and they form pseudoknot structures by base pairing, which is indispensable for its compact conformation that facilitates its functional significance in p53 signaling pathway [31,41]. Thus, the tertiary structures mainly attribute to the interactome of the lncRNAs, which further determines their cellular functional roles.

## 3. LncRNAs: Functional Mechanisms

With these abundant structural modifications, lncRNAs exhibit diverse mechanisms of action in regulating diverse cellular responses. Known mechanisms of action for lncRNAs at the molecular levels, broadly based on their sub-cellular localization, are discussed below.

### 3.1. Nuclear Functions

**Figure 2:**
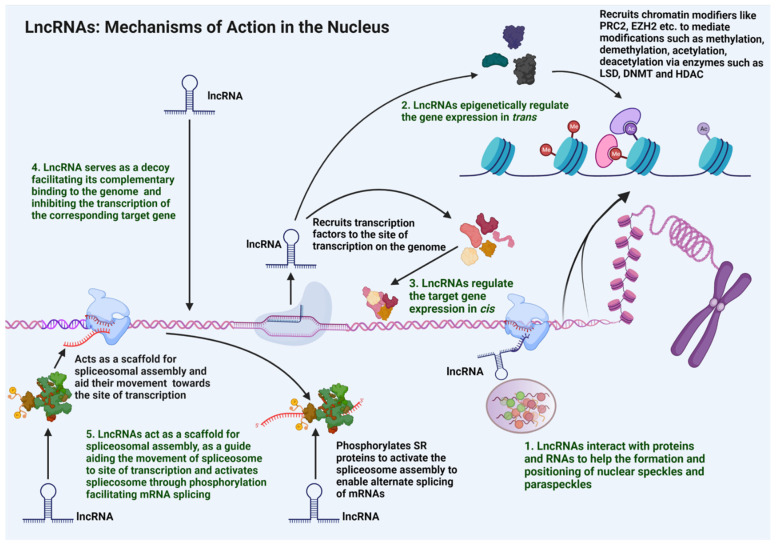
Mechanisms of nuclear lncRNA activity. The figure describes modes of action through which nuclear lncRNAs carry out their functions. (1) LncRNAs interact with several proteins and RNAs to aid the maintenance of nuclear architecture; (2) LncRNAs epigenetically regulate expressions of several genes by acting in *trans*; (3) LncRNAs regulate the gene expression by recruiting various transcription factors to the genetic locus; (4) LncRNAs also inhibit the gene expression by serving as decoy, through binding to the respective genetic loci directly; (5) LncRNAs serve as a scaffold for spliceosomal assembly as well as aid its movement across genetic loci to facilitate the mRNA splicing.

Within the nucleus, lncRNAs carry out functional roles such as modulation of nuclear architecture, epigenetic regulation of gene expression, regulation of transcription as well as mRNA splicing, dosage compensation, and genomic imprinting (Figure 2).

#### 3.1.1. Nuclear Architecture

LncRNAs located in the nucleus contribute to the maintenance of nuclear architecture [42]. They have been shown to be involved in the regulation of nucleosome positioning, chromosome positioning, and chromatin looping [42]. Such spatial regulation by lncRNAs contributes to the modulation of gene expression through transcriptional and post-transcriptional modifications of mRNAs in the nucleus that can impact their translation in the cytoplasm [42]. LncRNAs also play a major role in nuclear speckle formation [43]. Nuclear speckles are dynamic granular structures that are present in the inter-chromatin region and play a dominant role in the transcription of mRNA, especially in their splicing [43]. Functionally, nuclear speckles consist of splicing machinery inclusive of the spliceosomes, the serine/arginine rich (SR) factors, and snRNPs that translocate to the active regions of transcription to facilitate the mRNA splicing [43]. LncRNA MALAT1 is reported to associate with the nuclear speckle structures, acting as a scaffold to recruit the nuclear speckles to the active transcription sites for facilitating the mRNA splicing [44]. Nuclear paraspeckles are another sub-nuclear structure composed of RNA-protein structures present in the inter-chromatin region. They facilitate the nuclear retention of those specific mRNAs that have undergone either adenosine to inosine (A to I) editing or have several inverted repeats to form double-stranded RNA structures [45]. LncRNA NEAT1 is one of the key components of the nuclear paraspeckles. It interacts with proteins such as proline and glutamine rich splicing factor (PSF/SFPQ) and non-POU domain containing octamer binding protein (p54^nrb^/NONO) as well as paraspeckle component 1 (PSPC1/PSP1) protein to orchestrate the formation of nuclear paraspeckles. NEAT1 also helps in the positioning of the nuclear paraspeckles to the active transcription sites. This aids in the nuclear retention of double-stranded mRNAs that are formed out of the inverted repeats. Such retentions are reported to be associated with regulations in circadian rhythm, stress responses, viral infections, and pluripotency [46].

LncRNAs also take part in chromosome positioning. LncRNA FIRRE is located on both the X-chromosome and is transcribed even from the inactivated X-chromosomes, escaping the X-chromosome inactivation. FIRRE localizes the inactivated X-chromosome near the nucleolus, thereby helping the chromosome positioning [47]. LncRNAs aid chromatin looping to modify the gene expression. The lncRNA LUNAR1 is associated with the Notch occupied insulin-like growth factor 1 receptor (IGF1R)-enhancer regions as well as its own promoter through chromatin looping. This recruits the mediator molecules to the IGF1R target promoter to activate its expression [48,49].

#### 3.1.2. Dosage Compensation and Genomic Imprinting

LncRNAs also contribute to the dosage compensation as well as genomic imprinting in the cells. Dosage compensation is the process of inactivating one of the X chromosomes by silencing most of its gene expression in the female germline (XX) to compensate with the male germline (XY). One of the few genes that is transcribed and which is involved in dosage compensation is the lncRNA XIST. XIST is transcribed from the X-chromosome that undergoes inactivation. With the adenine-rich repeat domains, XIST transcript interacts with other proteins to promote the silencing of other genes on the X-chromosome, to inactivate them and result in dosage compensation. XIST can bind to PRC2/PRC1 complex to recruit DNA methyl transferases (DNMTs) and methylate the gene promoters to inactivate the expression of those genes. XIST can also bind to SMRT/HDAC1 associated repressor protein (SHARP), split end protein (SPEN), and silencing mediator for retinoid or thyroid hormone receptor (SMRT) protein complexes to recruit histone deacetylases (HDACs) to the genes, so as to suppress their expression [50,51,52].

Genomic imprinting labels gene expression to parental origin with modifications. LncRNA H19 is paternally imprinted and expressed from the maternal allele [53]. In addition, H19 takes part in imprinting of other genes by recruiting and interacting with methyl-CpG-binding domain protein 1 (MBD1). The H19-MBD1 complex recruits histone lysine methyl transferases to generate histone H3 lysine 27 trimethylation (H3K9me3) chromatin repression marks in their target gene loci such as those of insulin-like growth factor-2 (IGF2), solute carrier family 38 member 4 (SLC38A4), and paternally expressed gene 1 protein (PEG1), thereby suppressing the expression of either their paternal or maternal allelic genomic regions [53,54].

#### 3.1.3. Epigenetic Regulation

Nuclear lncRNAs play a critical role in chromatin remodeling through which the accessibility of chromatin regions to various components of transcriptional machinery and the resultant expression of specific gene loci are stringently regulated. LncRNAs can act as histone modifiers by modulating the activity of methylating-, demethylating-, or even acetylating-enzymes [55]. LncRNA HOTAIR inhibits the gene expression at homeobox (HOX) D cluster loci via PRC2 interaction, which is an example of chromatin repression [36]. HOTAIR interacts with PRC2, which facilitates H3K27me3-mediated repressive histone methylation through DNMTs. In breast cancer, HOTAIR is overexpressed and interacts with PRC2 to induce the repressive histone methylation, which suppresses the expressions of several tumor-suppressor genes, thereby resulting in tumor invasion and metastasis [37,56,57]. HOTAIR can mediate chromatin remodeling in PRC2 independent manner as well [58]. In human embryonic kidney cells as well as the kidney epithelial and mesenchymal cells, HOTAIR interacts with LSD1 to displace it from the promoters and enhancers of its target genes, thereby switching the normal epithelial phenotype to the mesenchymal one [59]. Several other lncRNAs also take part in regulating the methylome of the cancer cells, such as DACOR. In normal cells, the tumor suppressor lncRNA DACOR interacts with and activates DNMTs, resulting in repressive methylation of numerous oncogenes. This hypermethylation of the oncogene promoters reduces their expression, thus preventing any tumor initiation. Downregulation of DACOR, as seen in colon cancer, leads to the reduction of DNMT activity and subsequent hypomethylation of oncogene promoters that leads to the overexpression of oncogenes and tumor progression [56,57].

#### 3.1.4. Regulation of Transcription

LncRNAs regulate the transcription of various genes, by acting either as *cis*- or *trans*- elements. In the *cis*-mode, lncRNAs regulate the expression of genes in their respective chromosomal neighborhood. At least three different cis-regulatory mechanisms through which lncRNAs could modulate gene expression have been identified [60]. The first mechanism is where the lncRNA transcript directly mediates the recruitment of specific transcription factors so as to modulate gene regulation. For instance, during X-chromosome inactivation, XIST recruits specific gene silencing factors through its adenine repeat-rich domain to silence the genes present on the X chromosome [51]. The second mechanism is where the lncRNA directly modulates the expression of the neighboring gene. For example, the *IGF2R* (insulin like growth factor 2 receptor) gene, which is paternally imprinted, is associated with its anti-sense lncRNA, AIRN [61]. AIRN sequence spans part of the *IGF2R* gene as well as its promoter region. Thus, the binding of Airn transcript to *IGF2R* genomic locus silences the *IGF2R* expression [61]. LncRNA PANDA is antisense to the cyclin-dependent kinase inhibitor 1, p21 (*CDK1N1A*) gene that encoded cyclin-dependent kinase inhibitor, p21. Binding of PANDA to *CDKN1A* suppresses the p21 expression to promote cell proliferation [62]. The third mechanism is where the DNA sequence within the lncRNA loci can activate or repress the expression of genes in their vicinity. The lincRNA-p21, a TP53 induced lncRNA, is expressed in response to DNA damage. The lincRNA-p21 locus, whether it is being transcribed or not, activates the expression of the *CDKN1A* gene since the promoter regions of *CDK1N1A* lie within the lincRNA-p21 locus [63]. The enhancer lncRNAs (or e-lncRNAs), which arise from sequences within the enhancer regions of the neighboring genes, also take part in modulating the expression of genes in their vicinity. LncRNA PAUPAR represses the expression of the Paired Box gene 6 (*PAX6*) gene by acting in *cis*, since the PAUPAR locus contains certain enhancer elements for *PAX6*. Further, the PAUPAR-PAX6 level modulates several other enhancers, repressors, and promoter elements in executing transcriptional regulation of genes, so as to balance the cell cycle and modulate neural differentiation [64].

Several lncRNAs exhibit trans-regulatory mechanisms in controlling the expression of genes present in distal locations on the chromosomes. A classic example is provided by HOTAIR, which is transcribed from the *HOXC* locus. HOTAIR interacts with PRC2 and the lysine demethylase LSD1 to repress the gene expression at the distant HOXD locus, contributing to an epithelial–mesenchymal transition (EMT) in cancer cells [65,66]. A similar trans-regulatory mechanism of gene expression has been seen with lncRNAs such as MALAT1 and TUG1 [67].

### 3.2. Cytosolic Functions

**Figure 3:**
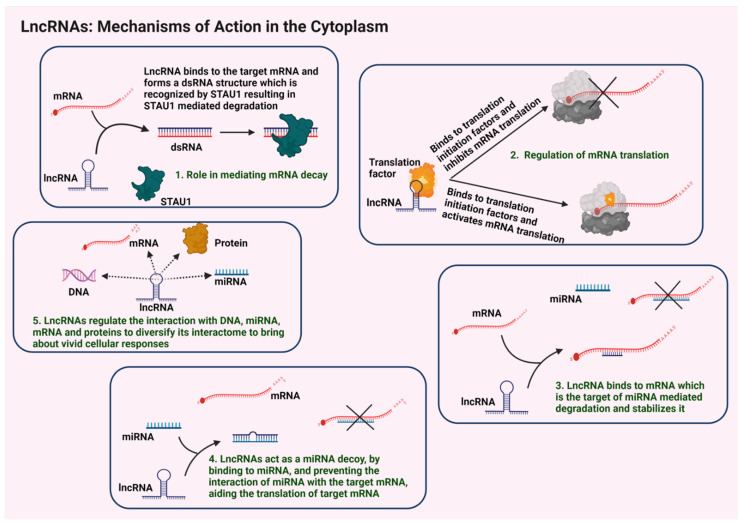
Mechanisms of cytoplasmic lncRNA activity. The figure describes the modes of action through which cytoplasmic lncRNAs carry out their functions. (1) LncRNAs contribute to different modes of mRNA degradation, thereby regulating the mRNA stability; (2) LncRNAs regulate mRNA translation by binding to the translation factors; (3) LncRNAs bind to mRNA and enhance their stability by preventing their miRNA-mediated inhibition; (4) LncRNAs bind to miRNAs and prevent their binding to the mRNAs; (5) LncRNAs also regulate the activity of their diverse interactomes that facilitate to carry out their functions within the cytoplasm.

The cytoplasmic lncRNAs play a major role in the regulation of both the transcriptional and translational machineries. Functions regulated by lncRNAs include the regulation of mRNA stability, translational regulations, sequestration of miRNAs, and functional modulation of proteins through specific interactomes (Figure 3).

#### 3.2.1. Regulation of mRNA Stability and Translation

For the maintenance of upregulated levels of cellular proteins, mRNA stability is a significant factor. LncRNAs play a major role in regulating the mRNA stability. It has been reported that the interactions of lncRNAs with numerous mRNAs either enhances their stability or promote their degradation [68]. In staufen, double-stranded RNA binding protein 1 (STAU1)-mediated mRNA decay, the STAU1 binds to its target mRNAs in its double-stranded form to initiate the degradation signals. The TINCR lncRNA binds to the STAU1 target mRNAs through the base pairing of the Alu repeats in both these RNAs, thereby forming a double-stranded RNA structure that in turn is degraded by the STAU1-mediated decay mechanism [69,70]. In detail, the TINCR binds with kruppel-like factor 2 (KLF2) mRNA, which stimulates the STAU1-mediated decay of KLF2 mRNA. This would adversely affect the expression of KLF2-regulated genes such as *CDKN1A* and *CDKN2B* that encode cyclin-dependent kinase inhibitors p21 and p15, respectively, leading to augmented tumorigenesis in gastric cancers [71,72]. LncRNAs also modulate the non-sense-mediated degradation (NMD) of mRNAs. Up-frameshift suppressor 1 homolog (UPF1) protein is a critical component of NMD pathway. LncRNAs SNHG6 and SNAI3-AS1 bind to UPF1 and recruit it to the mRNA that codes for the protein known as small mothers against decapentaplegic homolog 7 (SMAD7) and enables the degradation of SMAD7 mRNA in hepatocellular carcinoma via the NMD process. Since SMAD7 is a negative regulator of the TGF/SMAD pathway, this degradation promotes TGF-mediated EMT in these cancers [73].

LncRNAs also take part in regulation of translation within the cytoplasm by binding to several translation factors. LncRNA BC1 binds to the eukaryotic initiation factor 4A (eIF4A) and inhibits its helicase activity, thereby negatively regulating the mRNA translation [74]. LncRNA GAS5 interacts with translation initiation factor eIF4E and inhibits the translation of MYC gene that encodes c-Myc in lymphomas [75].

#### 3.2.2. Sequestration of miRNAs

Many of the cytosolic lncRNAs act as ceRNAs to sequester miRNAs that bind to and inhibit the target mRNAs, which possess corresponding miRNA response elements (MRE). Such lncRNA-miRNA binding suppresses the inhibitory action of miRNA on their target mRNAs, subsequently resulting in the expression of those genes targeted by the respective miRNAs. The lncRNAs that exhibit such sponging or inhibitory effect on specific miRNAs are termed as ceRNAs [76]. For example, under conditions of oxidative stress, the lncRNAs H19 and HULC act as ceRNAs by binding to miRNA let7a/b and miR-372/373 respectively, thereby inhibiting their activities. These interactions indirectly activate the expression of inflammatory cytokine interleukin-6 (IL6) as well as chemokine receptor type 4 (CXCR-4) that are targeted by these miRNAs. These inflammatory signals augment tumorigenesis in cholangiocarcinoma [77,78].

#### 3.2.3. Regulation of lncRNA-Specific Interactomes

Cytoplasmic lncRNA interactome includes numerous proteins. LncRNAs often play a role in regulating the activities of their interacting partners. For instance, pumilio RNA binding family member 1/2 (PUM1/2) proteins bind to specific mRNAs to trigger their degradation. However, the binding of the lncRNA NORAD to PUM1/2, reduces its availability to facilitate mRNA degradation [79]. Consistent with this functional role, PUM1/2 activity is increased upon the knockdown of NORAD, leading to the modulation of numerous mitotic regulators with the resultant chromosomal instability and aneuploidy [79]. With these aforesaid mechanistic roles, aberrant signaling by lncRNAs is critically involved in the genesis and progression of various human diseases, as discussed below.

A cautionary note here is that the functional roles of the lncRNAs have been deduced from the ectopic overexpression of the lncRNA or deletion of their expression. Differential localization of the overexpressed lncRNAs and compensatory activities of other lncRNAs in deletion experiments could contribute to erroneous conclusions. Therefore, complementary approaches are often required to assign a function to a specific lncRNA.

## 4. LncRNAs in Human Diseases

Recent findings on the structural and functional diversities of lncRNAs and their cognate interactomes have unraveled their potential role in the pathogenesis of many different human diseases. In addition to the structural and functional diversities that have been discussed in this review, we explore the divergent roles of lncRNAs in the induction and progression of major human diseases such as cardiovascular diseases, neurological disorders, cancer, and metabolic diseases such as diabetes. Major lncRNAs identified to play a role in these diseases are listed in Table 1.

### 4.1. LncRNAs in Cardiovascular Diseases

**Figure 4:**
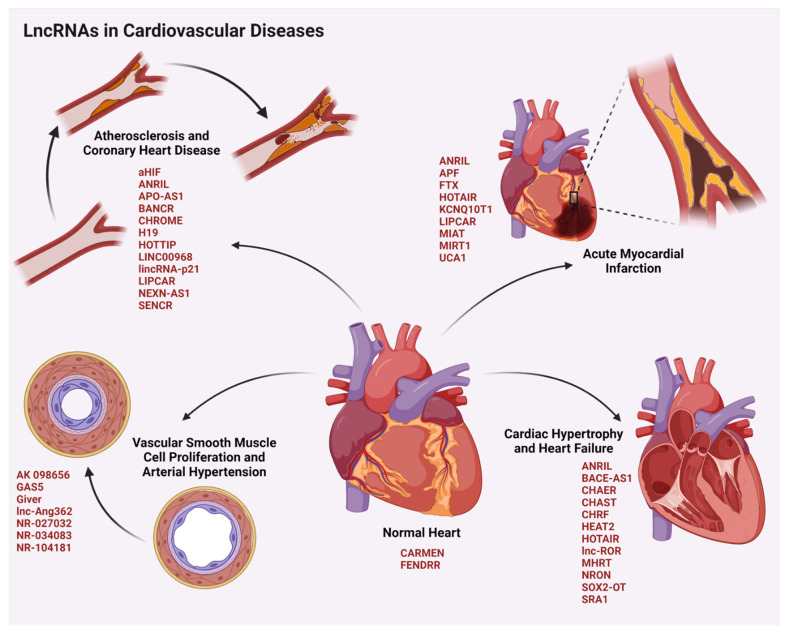
LncRNAs in cardiac physiology and pathology. The figure depicts the representative lncRNAs that aid in normal cardiac functioning as well as during cardiovascular complications such as arterial hypertension, atherosclerosis, coronary heart disease, acute myocardial infarction, cardiac hypertrophy, and heart failure.

Cardiovascular diseases (CVD) are the leading cause of mortality worldwide. The major stress responses result in either the autophagy, apoptosis, necrosis, or the hypertrophy of cardiomyocytes, leading to CVD. Several lncRNAs are expressed during diverse stages of development, differentiation, and maturation as well as pathogenesis of the cardiomyocytes (Figure 4).

LncRNAs expressed in the cardiac tissues mostly exhibit a cell/tissue-specific expression profile, which affects the cardiovascular development in the normal physiological conditions. For instance, lncRNA CARMEN regulates the cell fate, cellular differentiation, and homeostasis in human cardiac precursor cells [199]. Whereas the lncRNA FENDRR, which is expressed in the lateral mesoderm of the heart, is required for the heart wall development through its interactions with PRC2, trithorax group of proteins (TrxG), and mixed lineage leukemia protein (MLL) in modifying the chromatin state and gene expressions [200]. In addition to the normal developmental functions, lncRNAs also serve as a critical regulator of cardiovascular pathology, which includes conditions such as arterial hypertension, coronary heart disease, acute myocardial infarction, and heart failure [201].

#### 4.1.1. LncRNAs in Arterial Hypertension

Arterial hypertension (AHT) remains one of the most common forms of CVDs in addition to forming the basal triggering signal for other classes of CVDs. It arises mainly due to defects in proliferation, differentiation, and migration of vascular smooth muscle cells (VSMCs), which are the critical contractile elements of the blood vessel wall. In vivo experiments in rats with angiotensin-II (Ang-II) treatment revealed a role for the lncRNA lnc-Ang362. Overexpression of lnc-Ang362 elevates the expression of miR-221/222 leading to the activation of Nuclear factor kappa B (NF-κB) signaling. Enhanced NF-κB signaling promotes proliferation and migration of VSMCs to aggravate AHT [89]. Ang-II induces senescence of endothelial progenitor cells (EPCs), which play an important role in the repair of vascular endothelial damage, in hypertensive patients. The lincRNA-p21 was shown to protect EPCs from Ang-II damage by activating the SESN2/AMPK/TSC2 pathway and transcriptional activity of p53 that eventually enhanced autophagy. Another significant lncRNA that promotes AHT is Giver. In response to elevated Ang-II, the transcription factor known as nuclear receptor subfamily 4 group A member (NR4A3) is recruited to the promoter of Giver, which enhances the expression of Giver during AHT. Giver interacts with RNA Pol II and reduces the repressive histone H3K27me3 methylation of genes involved in oxidative stress such as *NOX1* that encodes NADPH Oxidase 1 as well as those involved in inflammation such as *IL6*, *CCL2*, and *TNF* (tumor necrosis factor), to promote their expression and thereby AHT [86]. LncRNAs that act as a scaffold to affect the protein stability and degradation also regulate AHT. For instance, lncRNA AK098656 interacts with 26S proteasomal components and facilitates the interaction of cytoskeletal VSMC specific contractile protein myosin heavy chain-11 with the proteasomal system to promote its degradation. This reduces the contractility of the vessels and enhancement of AHT [90]. Certain lncRNAs such as GAS5 are downregulated during AHT. Under normal conditions, GAS5 is expressed in endothelial cells (EC) and VSMCs. It regulates the vascular functions through β-catenin pathway. It acts as a ceRNA for miR-21 and elevates the expression of programmed cell death 4 (PDCD4) protein, a miR-21 target. PDCD4, in turn, attenuates AHT stimulation through the inhibition of platelet derived growth factor-bb (PDGF-bb)-induced VSMC proliferation and migration [96].

#### 4.1.2. LncRNAs in Coronary Heart Diseases

Several lncRNAs have been identified to be associated with coronary heart diseases (CHDs). The TNFα-induced lncRNA BANCR is overexpressed in VSMCs during CHD. It activates the c-Jun N-terminal kinase (JNK) pathway and promotes the proliferation and migration of VSMCs, which lead to atherosclerosis and CHD [81]. A similar effect has been exhibited by the TNFα/PDGF-bb-induced lncRNA HOTTIP, which is upregulated in CHD. However, this lncRNA enhances the proliferation and migration of the ECs, which is mainly through the Wnt-β-catenin signaling cascade [87]. The upregulation of lncRNA LINC00968 in response to enhanced levels of oxidized LDL (low density lipoprotein) enhances the proliferation and migration of the ECs to aggravate CHD by sequestering miR-9-3p [88]. In addition to the proliferation and migratory signals, metabolic reprogramming is also regulated by lncRNAs that promotes CHD. For instance, lncRNA CHROME, which is upregulated in CHD, is involved in the regulation of cellular and systemic cholesterol homeostasis. It is overexpressed in response to the higher levels of dietary cholesterol uptake through the activity of LXRs (Liver-X receptors). CHROME has also been identified to interact with argonaute-2 (Ago-2) protein that is involved in the sequestration or decay of its miRNA targets [85]. CHROME sequesters miR-27b/33a/33b/128, thereby upregulating the expression of miR-27b/33a/33b/128-target genes that encode cholesterol efflux transporter proteins such as ATP-binding cassette subfamily A member 1, a member of ATP-binding cassette transporters. These transporters aid cholesterol efflux from macrophages. The extruded cholesterol accumulates either in the tissues or deposit within the arterial walls causing atherosclerosis and CHD. In addition, CHROME-mediated sequestration of miRNAs leads to the expression of genes involved in HDL (high density lipoproteins) synthesis, which plays a pathogenic role in promoting atherosclerosis and CHD.

In contrast to the upregulation of pathogenic lncRNAs, lncRNAs with anti-atherosclerotic effects are downregulated in CHD. The antisense lncRNA, NEXN-AS1 is downregulated in atherosclerosis and CHD. NEXN-AS1 directly interacts with chromatin remodeling protein known as bromodomain adjacent to zinc finger domain 1A (BAZ1A) and induces the expression of *NEXN* gene that encodes nexilin F-actin binding protein. NEXN inhibits the oligomerization of Toll-like receptor-4 and the down-stream NF-κB signaling, which further inhibits the expression of inflammatory cytokines as well as cell adhesion molecules by ECs. This suppresses the adhesion of monocytes to the ECs and prevents atherosclerosis and CHD [101]. Likewise, the lncRNA SENCR, which is involved in the maintenance of the vascular endothelial cell integrity, is downregulated in CHD. SENCR maintains vascular EC adheren junctions through its interaction with cytoskeleton-associated protein 4. Although the mechanism underlying the downregulation of SENCR is not known, decrease in the expression of SENCR leads to defective EC differentiation and vascular permeability functions [102]. The downregulation of lincRNA-p21 has also been shown to be associated with atherosclerosis and CHD. LincRNA-p21, induced by p53, regulates the expression of several p53-downstream target genes, especially those involved in cell cycle arrest and apoptosis through its direct interaction with heterogeneous nuclear ribonucleoprotein K. LincRNA-p21 also interacts with mouse double minute 2 homolog protein, MDM2, to release its inhibitory effects on p53. Relieved p53 interacts with p300 and facilitates its recruitment to the promoter/enhancer sites of target genes in vascular smooth muscle cells [99]. Thus, the decreased expression of lincRNA-p21 in CHD results in enhanced cell proliferation and anti-apoptotic signaling along with the accelerated neointima formation in carotid arteries, all of which promotes atherosclerosis [99]. Some of the lncRNAs such as H19, ANRIL, and lincRNA-p21 exhibit genetic polymorphisms that enhance the risk of CHD in the patients [201].

#### 4.1.3. LncRNAs in Acute Myocardial Infarction

Acute myocardial infarction (AMI) results from the acute obstruction in the coronary artery due to atherosclerosis. The decreased blood flow due to atherosclerotic plaques in the coronary artery leads to reduced blood supply and oxygen to the myocardial tissues. This leads to myocardial ischemia as well as myocardial necrosis, and subsequently, an AMI or a heart attack. The pathological complications in patients are not limited to AMI incidence, but continues with the post-AMI difficulties such as cardiac fibrosis, ventricular remodeling, inflammatory responses, and ischemic–reperfusion injuries (IR injury) as well as apoptosis and autophagy of cardiomyocytes. Several lncRNAs are upregulated during the conditions of AMI that governs the complications post-AMI in patients. LncRNA MIAT is upregulated under conditions of AMI. It sequesters miR-24 and induces the expression of pro-fibrotic genes such as Furin and TGF-β1, which promotes proliferation of cardiac fibroblasts, accumulation of collagen and cardiac interstitial fibrosis. Thus, MIAT acts as a pro-fibrotic lncRNA, worsening the cardiac functions by promoting cardiac fibrosis during the post-AMI conditions [91]. Inflammation and apoptosis of the cardiomyocytes are the two worst cellular events that worsen the cardiac functions post-AMI and determine the fate of the heart. LncRNA MIRT1, which is upregulated in cardiac fibroblasts during AMI, has been reported to regulate the apoptosis of cardiomyocytes and myocardial inflammation. Silencing of MIRT1 in cardiac fibroblasts has been shown to inhibit the pro-apoptotic factors such as Bax, Bcl2, and caspases in cardiomyocytes. In vitro and in vivo studies have shown that knocking down MIRT1 can inhibit the macrophage infiltration into the myocardium thus attenuating the myocardial inflammation post-AMI. In addition, MIRT1 also promotes the NF-κB pathway that promotes the infiltration of inflammatory cells into the infarcted myocardium as well as peritoneal macrophage migration that results in worsening the post-AMI effects [92]. One of the post-AMI complications is the risk for ischemia–reperfusion (IR) injuries, in which the reperfusion of blood to the ischemic regions could cause tissue damages that can even lead to cardiac as well as multi-organ failures. IR injury is associated with enhanced oxidative and mitochondrial as well as endoplasmic stress that result in apoptosis of cardiomyocytes. LncRNA UCA1 overexpression has been reported to protect these cells from IR injury related endoplasmic reticulum stress and inhibit apoptosis in cardiomyocytes [94]. Furthermore, lncRNA APF (autophagy promoting factor), which is upregulated post-AMI, sequesters miR-188-3p and upregulates the expression of autophagy related 7 (ATG7) protein. ATG7 is critical for the autophagy of cardiomyocytes, thereby aggravating the injury after AMI [80]. In the same line, the lncRNA KCNQ1OT1 (KCNQ1 overlapping transcript 1), which is upregulated in MI patients and IR mouse models, was shown to promote autophagy-associated apoptosis of cardiomyocytes by sponging miR-26a-5p, which targets ATG12 [93].

Some of the lncRNAs such as HOTAIR and FTX are downregulated in post-AMI patients. HOTAIR sequesters miR-1 and regulates the expression of pro-apoptotic genes encoding B-cell lymphoma 2 apoptosis regulator, BCl2-associated X apoptosis regulator, and caspases to prevent apoptosis of cardiomyocytes [97]. FTX is also involved in cardioprotection through the inhibition of apoptosis in cardiomyocytes through the sequestration of miR-29b-1-5p to upregulate the expression of Bcl2-like 2 protein [95]. Thus, the downregulation of HOTAIR and FTX following AMI appears to accentuate myocardial injury.

#### 4.1.4. LncRNAs in Heart Failure

The terminal form of CVD is heart failure (HF), where the heart loses its ability to carry out its function of pumping blood across the human body. It arises as a result of the cumulative effects of other CVD attributes including the loss of ability of the heart to either contract or relax. In cardiomyocytes, elevated levels of lncRNA CHRF in response to Ang-II sequesters miR-489 and upregulates the expression of myeloid differentiation primary response 88 protein, encoded by *MYD88* gene. The latter induces cardiac hypertrophy and apoptosis in cardiomyocytes, leading to HF [84]. The lncRNA CHAER is also upregulated during HF. It interacts directly with PRC2, and prevents H3K27me3-repressive tri-methylation on target genes that promote cardiac hypertrophy such as those encoding MYH7, atrial natriuretic factor, and skeletal alpha actin, thereby augmenting mTOR signaling for HF [82]. Another significant lncRNA affecting cardiac hypertrophy and HF is CHAST, which is upregulated in cardiomyocytes upon receiving aortic constriction and HF signals. This negatively regulates the expression of the *PLEKHM1* gene that encodes pleckstrin homology domain-containing family M member 1 protein, which modulates autophagy and endocytic trafficking. Though the exact detailed mechanism is yet to be elucidated, the in vivo studies have shown that CHAST could promote cardiac remodeling and hypertrophy leading to HF [83]. Along with the increased expression of HF-promoting lncRNAs, a drastic downregulation was seen with MHRT, a cardioprotective lncRNA. MHRT is critically involved in the structural and functional homeostasis of the heart. Under normal physiological conditions, MHRT binds to brahma related gene 1 (BRG1) and inhibits its helicase activity. MHRT-BRG1 interaction prevents the action of BRG1/HDAC/poly ADP ribose polymerase (PARP) complex on chromatin modification of its target genes that alter the cardiac contractility, thus keeping the cardiac function intact. However, under conditions of extreme CHD and AMI leading to HF, MHRT is downregulated through the epigenetic repression by BRG1/HDAC/PARP complex on *MHRT* locus. Resultant dysregulation of the genes that were stringently regulated by MHRT along with BRG1-mediated cardiac remodeling leads to the loss of cardiac contractility and HF [100].

In addition to these causative lncRNAs that are being interrogated as therapeutic targets in CVDs, certain lncRNAs are being investigated as circulatory biomarkers for the different CVDs. First of its kind was the analysis of lncRNA LIPCAR levels in plasma, which was correlated with aberrant left ventricular remodeling post-AMI and enhanced risk of HF post-AMI [202,203]. In addition, several lncRNAs such as NRON, MHRT, ANRIL, BACE-AS1, HEAT-2, HOTAIR, linc-ROR, SOX-2-OT, and SRA1 have been investigated for their role in being biomarkers for HF [201]. The major circulatory lncRNA biomarker candidates for AHT includes GAS5, NR-027032, NR-034083, and NR-104181, while for CHD, it includes aHIF, APO-AS1, and LIPCAR [201]. Furthermore, ANRIL, KCNQ1OT1, ANRIL, and MIAT have been studied as lncRNA biomarkers in circulation for AMI [201]. Though the precise functional roles of these lncRNAs in cardiac functions are yet to be determined, further explorations on their mechanistic role in disease progression as well as their roles as biomarkers would undoubtedly bring them into clinical settings in the near future.

### 4.2. LncRNAs in Neurological Disorders

**Figure 5:**
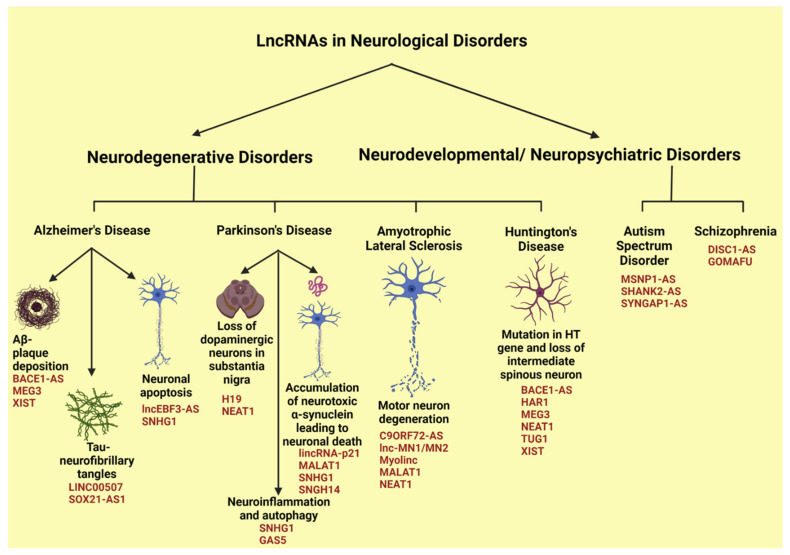
LncRNAs in neurological disorders: The figure represents lncRNAs involved in neurodegenerative disorders such as Alzheimer’s disease, Parkinson disease, amyotrophic lateral sclerosis, and Huntington’s disease, as well as the neurodevelopmental/neuropsychiatric disorders such as autism spectrum disorder and schizophrenia.

The central nervous system possesses the largest number of lncRNAs, which constitutes almost 40% of the total lncRNAs present in the human body [26]. They show greater conservation in the brain, along with the highest degree of spatial and temporal specificities than any other locations in the human body [104]. Under normal physiological conditions, lncRNAs regulate the development and differentiation of the neuronal cells and the nervous system. For instance, lncRNA DALI augments the expression of the *POU3F3* gene encoding the transcription factor POU class 3 homeobox 3 and acts as a cis-acting lncRNA. In addition, it forms a complex with POU3F3 to promote the activation of multiple genes to aid in neural differentiation as a trans-acting lncRNA [204]. On the contrary, BDNF-AS, an anti-sense lncRNA, binds to PRC2 and recruits the complex to the *BDNF* (brain-derived neurotrophic factor) gene locus to inhibit its expression. This adversely affects the BDNF-mediated axonal growth and differentiation [205]. Not just with the normal physiological conditions, lncRNAs execute various pathological roles as well, resulting in several neurodegenerative disorders [104,121]. These include diseases such as Alzheimer’s disease, Parkinson’s disease, amyotrophic lateral sclerosis, and Huntington’s disease, as well as neurodevelopmental/neuropsychiatric disorders such as autism spectrum disorder, and schizophrenia (Figure 5).

#### 4.2.1. LncRNAs in Alzheimer’s Disease

Alzheimer’s disease (AD) contributes to almost 50% of the dementia cases worldwide [206,207]. It is characterized by the deposition of β-amyloid plaques (Aβ-plaques) and the hyper-phosphorylation of Tau proteins that leads to neurofibrillary tangles, leading to neuronal inflammation as well as apoptosis and abrupt neuronal loss in the brain [104]. Aβ-plaques are formed from the amyloid precursor protein (APP), primarily by the action of beta site amyloid precursor protein cleaving enzyme 1 (BACE1). The antisense lncRNA BACE1-AS, which is highly upregulated in AD, binds to BACE1 and stabilizes it, to enhance the Aβ-plaque production [103]. LncRNA XIST, which is also highly expressed in AD, promotes Aβ-plaque formation. XIST sequesters miR-124 that targets BACE1, thus enhancing the expression levels of BACE1 and resultant Aβ-plaque formation [120]. Hyper-phosphorylation of Tau proteins accelerates the pathogenesis of AD as the Tau aggregation results in neurotoxicity and resultant loss of neuronal function, in addition to its role in promotion of Aβ plaque formation. LINC00507, which is upregulated in AD, promotes hyper-phosphorylation of Tau proteins by activating glycogen synthase kinase 3 beta (GSK3β) signaling cascade. It sequesters miR-181c-5p to enhance the expression of microtubule associated protein tau (MAPT), the gene encoding Tau protein, as well as tau tubulin kinase 1 (TTBK1) that phosphorylates Tau [108]. In addition, lncRNA SOX21-AS1 has also been reported to be upregulated in conditions of Aβ 1–42 induced AD pathogenesis. Though the exact downstream targets are not yet elucidated, it sequesters miR-107 to enhance Tau phosphorylation as well as promotes the neuronal apoptosis [118]. Interestingly, lncRNA SOX21-AS1 is currently investigated as a biomarker for AD [118].

Neuronal apoptosis is one of the prominent downstream effects of Tau phosphorylation and Aβ plaque deposition that worsens the AD pathogenesis. LncRNA SNHG1 is upregulated in AD and is induced by Aβ 25–35 deposition. It sequesters miR-137 and upregulates the expression of kringle containing transmembrane protein 1, which induces neuronal apoptosis that aggravates the AD pathogenesis [114]. Further, neuronal apoptosis is also enhanced by upregulated levels of lnc-EBF3-AS, which enhances the expression of its downstream target early B-cell transcription factor 3 (EBF3). EBF3 induces apoptosis of neurons in AD through upregulation of caspase activity and Bax proteins, while downregulating the BCL2 level [106]. Certain lncRNAs such as MALAT1 are downregulated in AD as well [123]. MALAT1 sequesters inflammatory miRNAs such as miR-125b and miR-155. Further, it enhances the expression of interleukin (IL)10 while downregulating the expressions of IL1β and TNFα. Furthermore, it alters the Janus kinase-signal transducer and activator of transcription (STAT), NF-κB, JNK, and p38 mitogen activated protein kinase pathways to modulate neuro-inflammation associated with AD [123]. In addition to MALAT1, lncRNA MEG3 is also downregulated in AD. Overexpression of MEG3 has been shown to reduce Aβ 25–35 deposition and the oxidative stress along with reduction of inflammatory signals through downregulation of IL1β, IL6, and TNFα as well as inhibition of Phosphoinositide 3-kinases (PI3K)/protein kinase B (Akt) signaling cascades in the hippocampus tissues of AD rat models [124]. Thus, the downregulation of MEG3 can be correlated with neuro-inflammation associated AD.

#### 4.2.2. LncRNAs in Parkinson’s Disease

Parkinson’s disease (PD) is another progressive neurodegenerative disease affecting elderly people characterized by loss of dopaminergic neurons in the substantia nigra of the brain. This disease condition is also characterized by the accumulation of α-synuclein, a pre-synaptic neuronal protein which in its abnormal insoluble form is neurotoxic, resulting in neuronal death [104]. Two major lncRNAs, namely H19 and NEAT1, have been demonstrated to be associated with loss of dopaminergic neurons in PD. The expression levels of H19 are reduced in PD. LncRNA H19 sequesters miR-301b-3p and upregulates the expression of hypoxanthine phosphoribosyl transferase 1 (HPRT1) as well as tyrosine hydroxylase. These enzymes activate Wnt/β-catenin signaling pathway through upregulation of genes such as *NGN2*, *NURR1* (nuclear receptor related protein 1), *PITX3* (pituitary homeobox 3), and *NEUROD1* (neuronal differentiation1), that prevent the loss of dopaminergic neurons. Therefore, the downregulation of H19 in PD results in the attenuation of this pathway and loss of dopaminergic neurons in PD [122]. Increased levels of α-synuclein are associated with the etiology as well as the progression of PD including the loss of dopaminergic neurons associated with PD. The elevated levels of SNHG14 sequester miR-133b to enhance the expression of α-synuclein, a miR-133b target [117]. In addition to promoting the expression levels of α-synuclein, SNHG1 can also promote its oligomerization. SNHG1 sequesters miR-15b-5p and enhances the expression levels of SIAH-family E3 ubiquitin protein ligase 1 (SIAH1), which further interacts with ubiquitin-conjugating enzyme H8 (UbcH8), an ubiquitin binding enzyme. SIAH1-UbcH8 ubiquitinates α-synuclein and promotes its aggregation, thereby worsening PD [115]. By increasing the expression and oligomerization of α-synuclein, SNHG1 plays a pivotal role in PD pathology. Several other lncRNAs also promote PD progression by modulating the expression and/or oligomerization of α-synuclein. LncRNA MALAT1, which is observed to be elevated in PD, binds to and enhances the α-synuclein stability, aggravating PD [110]. Further, the upregulated levels of lincRNA-p21 in PD sequesters miR-1277-5p and augments α-synuclein expression and aggregation, which contribute to the pathogenesis of PD [109].

Similar to AD, neuro-inflammation also worsens PD as it results in release of pro-inflammatory cytokines leading to neuronal apoptosis. The major lncRNAs affecting neuro-inflammation in PD are SNHG1 and GAS5, which act through miRNA sequestration. SNHG1 acts through miR-7 while GAS5 acts through miR-223-3p to upregulate NLRP3 (NOD-like receptor protein 3), which is involved in promoting inflammatory response in microglial cells [107,116]. In addition to neuro-inflammation, autophagy also contributes to aggravating the PD [208]. LncRNA SNHG1, which is upregulated in PD, sequesters miR-221/222 and indirectly regulates p27/mammalian target of rapamycin (mTOR) as well as microtubule associated proteins 1A/1B light chain 3B (LC-III) autophagic regulator expressions to inhibit autophagy, while promoting neuronal cytotoxicity in PD [209].

In comparison to other upregulated lncRNAs whose overexpression worsens the PD, elevated levels of NEAT1 exhibits neuroprotective roles during PD. NEAT1 is associated with nuclear paraspeckle formation and its upregulation is correlated with larger number of nuclear paraspeckles in dopaminergic neurons during PD. Under conditions of PD, these dopaminergic neurons result in loss of mitochondria and elevated oxidative stress that can damage the neuronal cells. However, NEAT1-mediated nuclear paraspeckle formation entraps numerous RNA and protein molecules within these structures, serving as a protective mechanism to the oxidative stress-mediated neuronal cell death. It has also been noted that NEAT1-assembled nuclear paraspeckles can entrap LRKK2, which mediates oxidative stress-mediated neuronal cell death. Interestingly, expression levels of NEAT1 are correlated further with gender-based incidence of PD. It has been observed that PD is less frequent in women. Since higher levels of estrogen induce NEAT1 that prevent the loss of dopaminergic neurons through its neuroprotective effects, the low incidence of PD in women has been correlated with estrogen-stimulated increase in the expression of NEAT1 [112]. However, this needs to be validated further with more experimental and epidemiological studies.

#### 4.2.3. LncRNAs in Amyotrophic Lateral Sclerosis

Amyotrophic lateral sclerosis (ALS) is a neurodegenerative disorder which affects the motor neurons in the brain stem, spinal cord, and motor cortex. The major gene mutations associated with ALS involve the genes *TDP43* and *C9ORF72* that encode transactive response DNA binding protein 43 and chromosome 9 open reading frame 72 protein, respectively. LncRNA NEAT1 serves as a scaffold for the interaction between RNA binding protein, TDP43 and fused in sarcoma/translocated in liposarcoma (FUS/TLS) protein, which forms the augmented number of accessory spots seen within the motor neuron nuclei, a property exhibited by the ALS patients [210]. Other lncRNAs that interact with TDP43 resulting in the pathogenesis of PD include MALAT1, Myolinc, and lnc-MN1/MN2, whose detailed mechanism of action are yet to be analyzed [104]. With regard to *C9ORF72* gene, GGGGCC (G4C2) repeat expansions at 5′ UTR of the gene results in not only the loss of function of the native protein, but also the generation of toxic proteins which aggravates ALS [105]. The antisense lncRNA, C9ORF72-AS has been identified to interact with the C9ORF72-mRNA. The abnormal GC repeats forms G-quadruplex structures that act as toxic molecules during ALS [105]. However the precise role of the interaction between C9ORF72-AS and C9ORF72-mRNA in the pathogenesis of ALS has not yet been elucidated [105].

#### 4.2.4. LncRNAs in Huntington’s Disease

Huntington’s disease (HD) is an autosomal dominant neurodegenerative disease characterized by abnormal CAG repeats in the *HTT* gene that encodes the Huntington protein, as well as selective loss of intermediate spinous neurons in the striatum. The mutant HTT proteins promote neurodegeneration mainly through dysregulated transcription, misfolded proteins, and oxidative stress as well as dysfunctional mitochondria [104]. The major lncRNAs upregulated in HD are MEG3, XIST, BACE1-AS, TUG1, and NEAT1, whereas the downregulated lncRNAs include HAR1. These lncRNAs have been reported to enhance the levels of mutant HTT protein, thereby aggravating the HD. However, the functional mechanism has not yet been identified [104].

#### 4.2.5. LncRNAs in Autism Spectrum Disorder

Autism spectrum disorder (ASD) is a neurodevelopmental disorder that is heterogeneous in genetic defects, ranging from single nucleotide variants to chromosomal abnormalities. The disease exhibits varied behavioral patterns, defective communicative skills as well as reciprocal social disconnections [121]. Major lncRNAs associated with ASD are the antisense lncRNAs such as SYNGAP1-AS, MSNP1-AS and SHANK2-AS, all of which are upregulated in ASD. The SYNGAP1-AS locus falls within the parent gene *SYNGAP1*, which codes for synaptic Ras GTPase activating protein-1, a critical protein involved in synaptic function and cognition. SYNGAP-AS1 reduces the expression of SYNGAP1 affecting the impairment of cortical function in the ASD patients [119]. Similarly, the lncRNA MSNP1-AS inhibits the expression of moesin, a neuronal factor involved in nuclear architecture and immune response. Upregulated MSNP1-AS reduces moesin levels and inhibits moesin-regulated RhoA, Rac, and PI3K/Akt pathways. These effects adversely affect the morphology and function of neurites, thus contributing significantly to ASD pathogenesis [111]. The antisense lncRNA SHANK2-AS inhibits the expression of SH3 and multiple ankyrin repeat domains 2 protein (SHANK2) in ASD. SHANK2 is a postsynaptic scaffolding protein involved in the structural and functional organization of diverse signaling pathways involved in post-synapse formation and development. The attenuation of SHANK2-promoted proliferative signals along with the propagation of apoptotic signals in neuronal cells drastically reduces the number as well as the length of neurites often observed in ASD [113].

#### 4.2.6. LncRNAs in Schizophrenia

Schizophrenia (SZ) is a neuropsychiatric disorder characterized by delusions, psychosis, depression, and dysphoria. Though the causative factors are not well described, numerous genetic, epigenetic as well as environmental factors have been shown to influence the SZ pathogenesis [121]. The most widely studied lncRNA associated with SZ is MIAT (also known as GOMAFU), which is downregulated in SZ. It interacts with splicing factors encoded by the genes QK1 (quaking homolog 1) and SRSF1 (serine/arginine rich splicing factor 1) to regulate their splicing activities. Hence, the downregulation of MIAT critically affects the global splicing, leading to alternative splicing of different pre-mRNAs. GOMAFU also regulates the expressions of *DISC1* (disrupted in schizophrenia 1) and *ERBB4* (Erb-B2 receptor tyrosine kinase 4) genes. Dysregulated alternative splicing of *DISC1* and *ERBB4* mRNAs mediated by MIAT has been associated with SZ. In vivo studies have also linked the reduction of MIAT expression with behavioral changes observed in SZ [125]. LncRNA DISC1-AS is also downregulated in SZ, which results in augmented DISC1 expression. DISC1 is involved in neurotransmitter signaling, especially the dopamine trajectories, by serving as a scaffold protein with a diverse interactome. Consequently, downregulation of DISC1 contributes SZ pathogenesis [121]. Thus, a greater in-depth analysis of the role of lncRNAs in regulating the gene expressions as well as cellular architecture and signaling can shed light on the link between the developmental and degenerative neurological disorders, which can be employed for tailored RNA-based therapeutic approaches.

### 4.3. LncRNAs in Cancer

**Figure 6:**
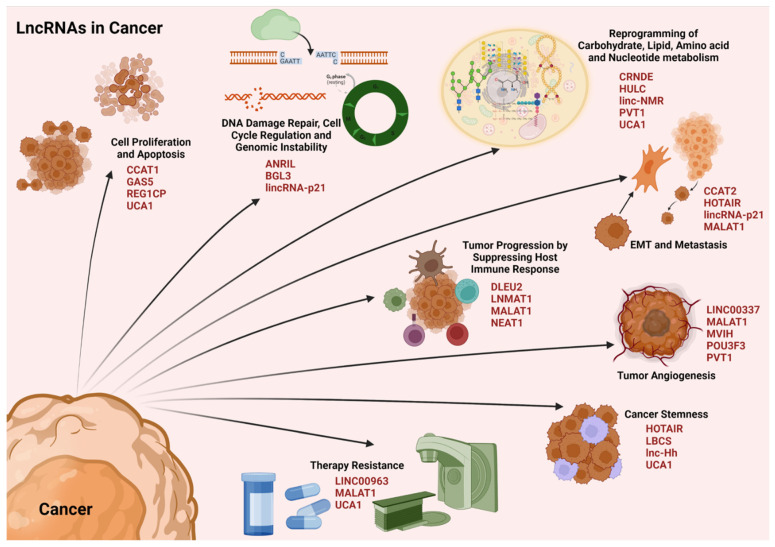
LncRNAs regulating tumorigenesis. The figure depicts the mechanisms by which representative lncRNAs regulate diverse cancer phenotypes such as cell proliferation and apoptosis, genomic instability, DNA damage repair, metabolic reprogramming, epithelial–mesenchymal transition and metastasis, host immune responses, tumor angiogenesis, cancer stemness, and therapy resistance.

Cancer has been defined as a pathological condition characterized by an uncontrolled proliferation of the cells, disrupting the tissue homeostasis. Major hallmarks of cancer include the self-sufficiency of growth signals, insensitivity of anti-growth signals, evading apoptosis, sustained angiogenesis, limitless replicative potential, tumor invasion and metastasis, reprogramming of energy metabolism, and the evasion of immune responses [211,212]. Recent studies have unequivocally demonstrated that noncoding RNAs play a cardinal role in modulating these cancer phenotypes through both genetic and epigenetic mechanisms [213,214,215]. Specifically, several lncRNAs have been identified as oncogenes while many others have been identified as tumor suppressors [213,216]. An in depth analysis of lncRNAs and their critical role in tumorigenesis has been recently reviewed [213]. Therefore, only a brief outline on the mechanism by which lncRNAs regulate diverse oncogenic phenotypes is presented here (Figure 6).

#### 4.3.1. LncRNAs and Cell Proliferation

During normal physiological conditions, there exists an equilibrium between cell proliferation and apoptosis that maintains the cellular homeostasis. However, the oncogenic signals enhance the pro-mitotic as well as anti-apoptotic signaling, that favor the aberrant cell proliferation in cancers. Tilting the cellular homeostatic balance towards cell survival and proliferation is carried out by lncRNAs through multiple mechanisms. The LncRNA UCA1, which is upregulated in gastric cancers, recruits EZH2 (enhancer of zeste homolog 2) to the promoters of the genes that encode p27 (*CDKN1B*) and the sprouty RTK signaling antagonist 1 (*SPRY1*) to mediate the repressive H3K27me3-trimethylation. This suppresses the expression of *CDKN1B* and *SPRY1* along with their tumor-suppressive activities, thus promoting gastric cancer cell proliferation [150]. In contrast to the epigenetic regulation of gene expression, lncRNA REG1CP, which is overexpressed in colorectal cancers, recruits FANCJ (Fanconi anemia of complementation group J) helicase at the *REG3A* (regenerating family member 3 alpha) promoter region. This association promotes the unwinding of DNA at the genetic locus of *REG3A* to promote its transcription, thus facilitating colon cancer cell proliferation [149]. In acute myeloid leukemia, lncRNA CCAT1 sequesters miR-155 to upregulate the downstream target c-Myc and subsequent signaling to augment cell proliferation [128]. The tumor suppressor lncRNA GAS5 is downregulated in triple negative breast cancers. GAS5 sequesters miR-196a-3p to elevate the expression of the transcription factor forkhead box O1 (FOXO1) expression, which inhibits the PI3K/Akt associated oncogenic signaling [155]. The downregulation of GAS5 in TNBC cell has an opposite effect with the resultant increase potentiation of PI3K/Akt-mediated oncogenic signaling.

#### 4.3.2. LncRNAs and Genomic Instability

Genomic instability and defective DNA damage response pathways are hallmarks of cancers. The scaffolding function of lncRNAs plays a primary role in the modulation of genomic instability in cancers. LncRNA ANRIL interacts with the DNA damage sensing protein ATR (ataxia telangiectasia) and prevents its ubiquitination. Thus, the homologous recombination-aided DNA double strand break repair remains intact in cancer cells facilitating uninterrupted cancer cell proliferation [126]. LncRNA BGL3 acts as a scaffold for BRCA1 (breast cancer type 1 susceptibility protein)/BARD1 (BRCA1 associated ring domain 1) and interacts with poly (ADP-ribose) polymerase 1 or PARP1 in facilitating a homologous recombination repair. This lncRNA has been investigated as a therapeutic target due to its depletion has been reported to sensitize cancer cells to therapeutic approaches that induce DNA damage [127]. One of the tumor-suppressor lncRNAs affecting genomic instability is the lincRNA-p21, which is downregulated in cancers. Under normal conditions, lincRNA-p21 interacts with heterogeneous nuclear ribonucleoprotein (hnRNP)-K and activates p53-mediated p21 expression, which further regulates the expression of various genes involved in cell cycle checkpoint regulation. This regulation is disrupted in cancers due to downregulated levels of lincRNA-p21 [157].

#### 4.3.3. LncRNAs and Metabolic Reprogramming

Metabolic reprogramming of the cells is a cardinal facet during oncogenic transformation as well as cancer progression. The changes occur in carbohydrate, lipid, amino acid, and nucleotide metabolisms in the cancer cells. The major source of energy for the cancer cells is glucose, and the proliferating cancer cells utilize glucose mainly through the time-efficient aerobic glycolysis instead of the energy-efficient mitochondrial oxidative phosphorylation, well explained as the Warburg effect. Numerous lncRNAs promote glucose metabolism either by enhancing levels of glucose transporters, as for instance, lncRNA CRNDE that upregulates GLUT1 (glucose transporter 1) expression in colorectal cancers, or through upregulating enzymes in the aerobic glycolytic pathway, as for instance, lncRNA PVT1 that enhances hexokinase levels by sequestering miR-143 [130,147]. Lipid metabolism is also significant in tumorigenesis as lipids act as membrane components, signaling molecules as well as energy source. For instance, lncRNA HULC is upregulated in hepatocellular carcinoma where it sequesters miR-9 and elevates peroxisome proliferator-activated receptor alpha (PPARA) levels. The transcription factor PPARA further binds to the promoter of *ACSL1* (acyl-CoA synthetase long chain family member 1) gene and transactivates its expression, which is a major subunit of acetyl CoA synthase involved in triacylglycerol synthesis [132]. The major amino acid whose metabolism is altered in cancer cells is glutamine, as it serves as the source for glutamate and α-ketoglutarate by the action of glutaminase (GLS) enzyme. LncRNA UCA1, which is upregulated in bladder cancers, promotes glutaminolysis through enhancing the expression of GLS2, which codes for glutaminase enzyme via miR-16 sequestration [151]. The studies on lncRNAs affecting nucleotide metabolism are very scarce. In hepatocellular carcinoma, the elevated levels of linc-NMR has been reported to interact with Y-box binding protein 1 (YBX1) and regulate the enzymes in pyrimidine metabolism such as ribonucleotide reductase regulatory subunit M2 (RRM2), thymidylate synthetase (TYMS), and thymidine kinase 1 (TK1) to enhance the deoxynucleotide triphosphate availability, favoring cancer cell proliferation [135].

#### 4.3.4. LncRNAs and Immune Evasion

Immune suppression is one of the major pre-requisites for cancer cell proliferation and progression. The immune response in the human body occurs either through adaptive or innate mechanisms. The adaptive mechanism employs either activated T-cells or B-cells to evoke immunity of a previously recognized pathogen or pathogenic state. Activated CD8+ cytotoxic T lymphocytes kill the cancer cells through the release of TNFs and ILs, besides other mechanisms.

The upregulation of lncRNA NEAT1 promoted CD8^+^ T cell apoptosis via the sequestration of miR-155 and consequent increase in Tim-3 (T cell immunoglobulin and mucin domain containing protein 3). This results in immune evasion and the growth of hepatocellular carcinoma [145]. LncRNA MALAT1 sequesters miR-195 to enhance the expression of programmed death ligand 1 (PDL-1) expression, which facilitates the apoptosis of cytotoxic T-lymphocyte apoptosis and immune escape, favoring tumorigenesis [140]. LncRNAs play a major role in the regulation of B-cell-mediated humoral immune responses as well. For instance, lncRNA DLEU2 sequesters miR-15a/16 to regulate B-cell proliferation through the upregulation of its downstream target genes involved in proliferation such as *CCND2/3* and *CCNE* in chronic lymphocytic leukemia [131]. Major immune cells associated with the innate immune responses are the macrophages, natural killer (NK) cells, and myeloid derived suppressor cells (MDSCs). The M2 polarization of macrophages promotes the tumor progression by recruiting MDSCs in the tumor microenvironment, thus suppressing the immune responses. LncRNA LNMAT1 recruits hnRNPL to the promoter of *CCL2* gene to activate its expression through H3K4-me3 trimethylation. Subsequent CLL2-promoted infiltration of M2-type tumor associated macrophages and the upregulation of CCL2-responsive genes enhance cancer progression through immune evasion in bladder cancers [139].

#### 4.3.5. LncRNAs and Epithelial–Mesenchymal Transition

EMT and metastasis form the critical phases in tumor progression. LncRNA HOTAIR interacts with PRC2 and induces H3K27me3-mediated repressive methylation on the promoter of tumor suppressive genes such as *PTEN* (phosphatase and tensin homolog) and *JAM2* (junctional adhesion molecule B). It can also interact with LSD1 to induce H3K4-demethylation to activate the gene expression on the promoter of *LDHA* (lactate dehydrogenase A) and CCNA1 (cyclin A) genes, thereby promoting EMT and metastasis [133]. LncRNA MALAT1 sequesters miR-126-5p and enhances the expression of Slug and Twist to facilitate EMT and metastasis in colorectal cancers [141]. By acting as a scaffold, lncRNA CCAT2 interacts with the transcription factor termed transcription factor 7-like 2 (TCF7L2). TCF7L2 transactivates the expressions of c-Myc and oncogenic miRNAs such as miR-17-5p and miR-20a, thus aiding metastasis in colorectal cancers [129]. Several tumor suppressor lncRNAs that affect EMT and metastasis are downregulated in cancers, one of these being lincRNA-p21. Normally, lincRNA-p21 sequesters miR-9 to upregulate the expression of E-cadherin, a suppressor of EMT. However, the downregulation of lincRNA-p21 relieves this inhibition on miR-9 and the ensuing downregulation of E-cadherin contributes to tumor invasion in hepatocellular carcinoma [158].

#### 4.3.6. LncRNAs and Tumor Angiogenesis

Angiogenesis is significant for cancer genesis and progression as it aids nutrient and oxygen supply to the proliferating cancer cells. Increase in VEGFA levels mediated by STAT3-signaling plays a critical role in tumor angiogenesis. LncRNA PVT1 enhances the stability of STAT3 by preventing it from ubiquitin-mediated proteolysis, thus promoting tumor angiogenesis [148]. In hepatocellular carcinoma, lncRNA MVIH inhibits the secretion of phosphoglycerate kinase 1 (PGK1) through a direct interaction, which prevents the inhibitory effects of PGK1 on tumor angiogenesis. PGK1 acts as a disulphide reductase that reduces the disulfides in protease plasmin, which in turn promotes the release of the angiogenic inhibitor angiostatin [144]. In breast cancer, lncRNA MALAT1 sequesters miR-140 in order to upregulate VEGFA expression to promote tumor angiogenesis [142]. Regulation of tumor angiogenesis by lncRNAs also involves epigenetic mechanisms. LINC00337 recruits DNMT1 to the promoter of the tumor-suppressive gene, *CNN1* (calponin 1), to repress its expression in colorectal cancers, which further promotes VEGF-mediated tumor angiogenesis [136]. LncRNAs also regulate the tumor microenvironment to promote tumor angiogenesis. The exosomal lncRNA POU3F3 released by glioma cells upregulates VEGFA and bFGF (basic fibroblast growth factor) in endothelial cells, thus promoting angiogenesis [146].

#### 4.3.7. LncRNAs and Cancer Stemness

Cancer stemness is the key feature that contributes greatly to cancer relapse and therapeutic resistance. LncRNA-Hh promotes sonic hedgehog–glioma-associated oncogene homolog 1 pathway to upregulate the expression of transcription factors, SRY-related HMG box transcription factor 2 (Sox-2) and octamer-binding transcription factor 4 (Oct-4), to enhance the self-renewal of cancer stem cells and mammosphere formation in breast cancers [138]. In cervical cancers, the elevated levels of UCA1 sequester miR-122-5p to upregulate Sox-2 expression, thereby facilitating cancer stemness [152]. LncRNA HOTAIR recruits PRC2 to the promoter of gene encoding miR-7 to induce repressive H3K27me3 methylation. Further, the upregulation of its downstream targets such as c-Myc and Twist through miR-7/SETDB1 (SET domain bifurcated histone lysine methyl transferase 1)/STAT3 axis enhances the stemness in breast cancers [133,134]. The tumor suppressor lncRNA LBCS is downregulated in bladder cancers, which otherwise recruits hnRNPK and EZH2 to the promoter of *SOX2* gene to induce H3K27me3 repressive tri-methylation, thus suppressing *SOX2* expression [156].

#### 4.3.8. LncRNAs and Therapy Resistance

LncRNAs regulate therapy resistance in cancers through different mechanisms. LncRNAs MALAT1 and LINC00963 contribute to radioresistance in nasopharyngeal cancers and breast cancers, respectively. While the former acts via miR-1/Slug axis, the latter acts through miR-324-3p/activated Cdc42 associated kinase1 (ACK1) axis to enhance radioresistance [137,143]. Slug, which is an oncogenic transcription factor, and ACK1, which is an oncogenic receptor tyrosine kinase, promote downstream signaling facilitating radioresistance in these cancers. LncRNA UCA1 sequesters miR-27b and upregulates *CCNG1* (cyclin G1) expression, which subsequently elevates p53 level, and downregulates miR-508-5p to induce multidrug resistance in gastric cancers [153,217]. UCA1 also recruits EZH2 to the promoter of *CDKN1A/p21* gene to suppress its expression and activate the PI3K/Akt pathway, thereby contributing to tamoxifen resistance in breast cancers [218]. Certain lncRNAs also upregulate the drug efflux transporters to induce drug resistance in cancers. LncRNA MALAT1 upregulates multidrug resistance protein 1 (MRP1) and multidrug resistance 1 (MDR1) proteins to enhance drug efflux and, thereby, inducing multidrug resistance in lung cancers [219].

An in depth analysis of the functional associations of different lncRNAs with the different cancers and cancer sub-types could identify them as druggable targets for cancer drug discovery and tailored cancer therapy.

### 4.4. LncRNAs in Diabetes Mellitus

**Figure 7:**
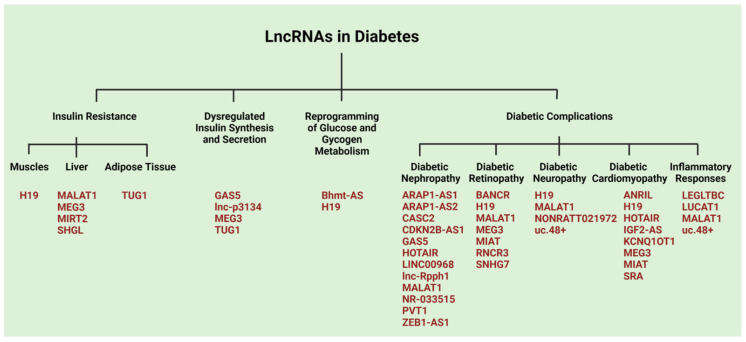
LncRNAs in diabetes mellitus. Major lncRNAs affecting the diverse phenotypes of diabetes mellitus such as insulin resistance, deregulated insulin synthesis and secretion, reprogramming of glucose, and glycogen metabolisms are depicted. In addition, lncRNAs involved in diabetic complications such as diabetic nephropathy, diabetic retinopathy, diabetic neuropathy, diabetic cardiomyopathy, and inflammatory responses are presented.

Diabetes is one of the most common metabolic disorders with chronic impacts. Diabetes is a broader term which encompasses the group of diseases that manifest impairment in glucose utilization [220]. In general, chronic diabetes is categorized as type 1 diabetes mellitus (T1DM) and type 2 diabetes mellitus (T2DM) whereas T1DM is characterized by defective insulin synthesis primarily due to pancreatic β-cell destruction whereas T2DM is characterized by progressive loss in insulin secretion along with insulin insensitivity [221,222]. Since T2DM comprises more than 90% of all diabetic cases, the role of lncRNAs in T2DM is discussed here. However, the correlates discussed here are likely to have an impact on prediabetes and gestational diabetes since both prediabetes and gestational diabetes often progress towards T2DM. LncRNAs are involved in regulating multiple pathological complications associated with DM (Figure 7).

Aberrant expression of lncRNAs can be attributed to insulin resistance, insulin synthesis, and reprogramming of glucose metabolism. In addition, dysregulated lncRNAs contribute significantly to the ailments associated with DM such as diabetic nephropathy, diabetic retinopathy, diabetic neuropathic pain, diabetic cardiomyopathy cardiovascular, and inflammatory complications [159].

#### 4.4.1. LncRNAs in Insulin Resistance and Synthesis

Several lncRNAs are associated with insulin resistance in DM. In the muscles, the major pathway involved in glucose uptake, fatty acid oxidation, and insulin sensitivity is the AMPK (5’ AMP-activated protein kinase) signaling cascade. LncRNA H19, which promotes AMPK signaling, is downregulated in DM. H19 inhibits let-7 expression, thereby promoting the expression of genes involved in insulin sensitivity such as *DUSP27* (dual specificity phosphatase 27), *IDE* (insulin degrading enzyme), *INSR* (insulin receptor), and *IRS2* (insulin receptor substrate 2) [184]. In the muscles, downregulation of H19 contributes to insulin resistance by dysregulating the β-oxidation of fatty acids in mitochondria and accumulating in the fatty acids. Under normal physiological conditions, H19 recruits hnRNPA1 to the mitochondria, which promotes the translation of carnitine palmitoyl transferase 1 beta (CPT1B) and peroxisome proliferator-activated receptor gamma coactivator 1 alpha (PGC1A) mRNAs that are required for the β-oxidation of fatty acids [183]. LncRNAs have also been shown to regulate the hepatic glucose and lipid metabolisms, thereby contributing to hepatic insulin resistance and DM. The major transcription factor required for lipid biosynthesis in liver is sterol regulatory element-binding transcription factor 1c (SREBP-1c). LncRNA MALAT1, which is overexpressed in DM, binds to and stabilizes SREBP-1c protein to promote lipogenesis and hepatic insulin resistance in vivo [168]. LncRNA MEG3 contributes to hepatic insulin resistance in DM by sequestering miR-214 and upregulating the transcriptional factor ATF4 (activating transcription factor 4), which promotes gluconeogenesis through upregulation of glucose-6-phosphatase and phosphoenolpyruvate carboxy kinase enzymes [173]. In regulating gluconeogenesis, lncRNA SHGL recruits hnRNPL to promote calmodulin expression, that inhibits gluconeogenesis. However, SHGL is downregulated in DM, thus impairing gluconeogenesis in DM [194]. LncRNA MIRT2 sequesters miR-34-5p and upregulates its target ubiquitin specific peptidase 10 (USP-10). USP-10 interacts with sirtuin-6 to counteract hepatic insulin resistance. With the downregulation of MIRT2 in DM, this process is reversed and insulin resistance is promoted in DM [193]. In addition to muscles and liver, adipose tissue also exhibits insulin resistance in DM. LncRNA TUG1 sequesters miR-204 and augments SIRT1 expression, which enhances insulin sensitivity in adipose tissue through activated GLUT4/PPARγ/Akt signaling. The downregulation of TUG1 in DM leads to decreased insulin sensitivity [196].

LncRNAs also affect the pancreatic β-cell function as well as the insulin synthesis. The downregulated MEG3 levels in DM reduce the expression of transcription factors musculoaponeurotic fibrosarcoma oncogene family protein A (MAFA) and pancreatic and duodenal homeobox 1 (PDX1), which in turn inhibit insulin synthesis and secretion from the pancreatic β-cells [191]. Further, the reduced levels of lncRNA GAS5 in DM also adversely affect the MAFA and PDX1 expressions as well as cell cycle arrest to inhibit the pancreatic insulin synthesis [181]. LncRNA-p3134 promotes the expression of MAFA, PDX1, GLUT2, and TCF7L2 in pancreatic β-cells, which enhances insulin secretion in response to higher glucose levels. This points out to the protective role of lncRNA-p3134 from toxic effects of enhancing the rate of glucose-induced insulin secretion from pancreatic cells, while remaining elevated in DM [166]. Inhibition of lncRNA TUG1 in the pancreatic β-cells contributed to defective insulin synthesis through either promoting the β-cell apoptosis or through reduction in transcription factors such as MAFA and PDX1, which plays a role in insulin synthesis [197].

#### 4.4.2. LncRNAs in Glucose Metabolism

The role of lncRNAs in reprogramming glucose metabolism also contributes to DM. In DM, glucose levels are elevated in the liver due to defective glycolysis, and increased gluconeogenesis or glycogenolysis. This can be correlated with the downregulation of lncRNA H19 in DM. Downregulation of H19 enhances the expression level as well as nuclear localization of FOXO1 transcriptional factor. FOXO1 enhances the transcription of gluconeogenic genes thereby promoting hepatic glucose accumulation and DM pathogenesis [185]. Hepatic gluconeogenesis is also promoted by the lncRNA Bhmt-AS, which is upregulated in DM. Bhmt-AS, an antisense lncRNA, stabilizes the expression of betaine homocysteine S-methyl transferase enzyme, which is involved in hepatic gluconeogenesis [160]. In addition to modulating predisposing factors and signaling pathways promoting DM, numerous lncRNAs are also associated with microvascular and macrovascular complications associated with the disease, which includes diabetic nephropathy, diabetic retinopathy, diabetic neuropathic pain, diabetic cardiomyopathy, and inflammatory complications [159].

#### 4.4.3. LncRNAs in Diabetic Nephropathy

Diabetic nephropathy (DN) is one of the microvascular complications of DM damaging the renal tissue and functions. Primarily, the proliferation and fibrosis of the mesangial cells contribute to diabetic nephropathy. LncRNAs LINC00968 and GAS5 recruit EZH2 to the promoter of *CDKN1A* and *MMP9* genes (matrix metalloproteinase 9), respectively, to repress their expression. LINC00968 is upregulated while GAS5 is downregulated under conditions of DN. However while LINC00968-mediated downregulation of p21 promotes proliferation and fibrosis of mesangial cells through enhancing the expression of ECM proteins, thus aggravating DN, GAS5-mediated inhibition of MMP9 expression reduces the inflammatory signals to attenuate renal fibrosis and prevent the burden of DN [165,182]. LncRNA PVT1, which recruits EZH2 to the promoter of *FOXA1* to induce H3K27me3-mediated repressive tri-methylation, is overexpressed during conditions of DN. The lower FOXA1 levels, in turn, regulates apoptotic genes and facilitates podocyte apoptosis and nephropathy in DM [177]. LncRNA Rpph1 overexpression enhances its interaction with galectin 3 and activates galectin-3/mitogen-activated protein kinase kinase/extracellular signal-regulated kinase pathway aiding proliferation and inflammation of the mesangial cells [167]. LncRNA MALAT1 sequesters miR-145 and induces the expression of the transcription factor zinc finger E-box binding homeobox 2 (ZEB2) expression, which promotes the transcription of ECM genes aiding renal fibrosis [169]. Synergizing with this mechanism, lncRNA ZEB1-AS1 that suppresses renal fibrosis, is downregulated in DN. Normally, ZEB1-AS1 sequesters miR-217 and upregulates the transcription factor MAFB (musculoaponeurotic fibrosarcoma oncogene homolog B) along with reduction in ECM proteins such as fibronectin and collagen, to inhibit renal fibrosis in DM [198]. The downregulation of ZEB1-AS1 favors renal fibrosis in DM.

#### 4.4.4. LncRNAs in Diabetic Retinopathy

Diabetic retinopathy (DR) is another major complication associated with DM that damages the retinal cells and vision. LncRNA RNCR3, which is upregulated in DR, sequesters miR-185-5p and augments the expression of the transcription factor KLF2 to promote retinal endothelial cell proliferation, migration, and tube formation. This further leads to augmented acellular capillaries, increased vascular leakage and inflammatory responses that result in retinal reactive gliosis and DM induced retinal neurodegeneration [178]. LncRNA MALAT1 sequesters miR-125b to upregulate downstream target gene expressions, such as that of vascular endothelial cadherin, aiding proliferation and vascular permeability of retinal microvascular endothelial cells, to adversely affect the vision by promoting neovascularisation [170]. In contrast to these upregulated lncRNAs, levels of lncRNAs H19, SNHG7, MEG3, and BANCR are downregulated in DR. LncRNA H19 sequesters miR-93 to upregulate the expression of X-box binding protein 1, resulting in reduction of inflammatory cytokines and inflammation in retinal epithelial cells. Downregulation of H19 leads to the reversal of the inhibitory control in DR [186]. Similarly, the downregulation of SNHG7 relieves its inhibition of retinal endothelial cell angiogenesis via the miR-543-SIRT1-VEGF signaling axis [186,195]. Likewise, the downregulation of MEG3 releases its inhibitory control to prevent DR through the reduction of VEGF and TGF-β1 expressions in retinal cells [192].

#### 4.4.5. LncRNAs in Diabetic Neuropathic Pain

Diabetic neuropathic pain (DNP) is a prominent chronic complication of DM. LncRNA NONRATT021972 is upregulated in patients with DNP. It increases the expression of TNFα as well as the expression of purinergic receptors (P2X) 3 and 7 to aggravate the DNP conditions [176]. Elevated levels of lncRNA uc.48+ enhances P2X3 receptor expression to aggravate DNP. P2X3 signaling activates downstream ERK1/2 and TNFα to bring out excitatory transmission signals that contribute to DNP [179]. Another major symptom or disease complication associated with DM is the chronic wounds in the patients. LncRNA H19 has been reported to augment the wound healing process in DM. It activates fibroblasts through recruitment of EZH2 to induce H3K4me3 methylation on the promoter of *HIF1A* gene that encodes hypoxia inducible factor 1α promoter to augment its expression and fasten the wound healing process [187]. Thus, the reduced levels of H19 in DM contribute significantly to the impaired wound-healing process. Though lncRNA MALAT1 is overexpressed in DM, it has been shown that transplantation of MALAT1-overexpressing human mesenchymal stem cells improves the wound healing in DM patients. In these stem cells, MALAT1 sequesters miR-205-5p to augment VEGF levels to promote wound healing in DM [223].

#### 4.4.6. LncRNAs in Diabetic Cardiac Myopathy

Diabetic cardiac myopathy (DCM) is characterized by the presence of myocardial abnormalities leading to cardiac complications, such as heart failure, in patients with DM. Similar to the other allied complications of DM, the incidence and progression of DCM is also regulated by several lncRNAs. The lncRNA KCNQ1OT1, which is upregulated under conditions of DCM, has been shown to sequester miR-214-3p and alter caspase-1 expression in cardiomyocytes. This resulted in the pyroptosis-mediated death of cardiomyocytes, deregulated cytoskeletal structures, and enhanced calcium overload, all of which contribute to the worsening of cardiac functions [163]. LncRNA MIAT sequesters miR-22-3p to upregulate DAPK2 (death associated protein kinase 2) expression, resulting in the apoptosis of cardiomyocytes along with the worsening of the left ventricular function in diabetic animal models [175]. Furthermore, MEG3, which is upregulated in DCM, sequesters miR-145 to induce the expression of the apoptotic protein known as programmed cell death 4 (PDCD4). PDCD4 is a cardinal regulator of cellular apoptosis that alters BAX/BCL2 levels to induce cardiomyocyte apoptosis in DM [174]. LncRNA IGF2-AS inhibits IGF2 as well as VEGF expression in myocardial microvascular endothelial cells, thereby inhibiting angiogenesis, and affecting the cardiovascular system during DM [162]. In addition to these upregulated lncRNA, several lncRNAs that have cardioprotective roles are downregulated in DCM. In normal cellular homeostasis of cardiomyocytes, H19 downregulates DIRAS3 (distinct subgroup of the Ras family member 3) expression through the recruitment of EZH2 to induce H3K27me3 methylation on the *DIRAS3* promoter [188]. DIRAS3 inhibits cell proliferation and promotes autophagy-mediated cell dormancy [224]. Downregulation of H19 observed in DCM leads to the depression of DIRAS3 and the resultant inhibition of Akt-signaling pathway and autophagy of cardiomyocytes, promoting DCM [188]. Likewise, the downregulation of HOTAIR results in the reduced viability of cardiomyocytes through inactivation of PI3K/Akt signaling cascades [189].

#### 4.4.7. LncRNAs in Diabetes-Associated Inflammation

Several lncRNAs also modulate inflammatory signals that can directly impinge on the pathological complications associated with DM. LncRNA MALAT1 mediates the activation of NLRP3-mediated inflammation resulting in cardiovascular complications in DM [171]. The macrophage polarization also contributes to inflammatory responses associated with DM. The levels of circulating lncRNA uc.48+ are elevated in DM patients. It enhances the P2X7 receptor expression and elevates the levels of the pro-inflammatory cytokines, IL-1β and TNFα, promoting apoptosis and inhibition of the proliferation of macrophages during DM [179,180]. In the cases of cerebral IR injury associated with DM, MALAT1 worsens the condition through the activation of MyD88/IRAK1 (interleukin 1 receptor associated kinase 1)/TRAF6 (TNF receptor associated factor 6) signaling cascade resulting in an inflammatory response in microglia [172]. LncRNA LEGLTBC sequesters miR-34a and upregulates SIRT1 expression to promote apoptosis in INS-1 beta cells, thereby adversely affecting the insulin synthesis and release, aggravating DM [164]. In instances of diabetic lung disorders, lncRNA LUCAT1/SCAL1 is reduced in the serum considerably, which otherwise inhibits iNOS (inducible nitric oxide synthase) expression and nitric oxide production in the lung cells during DM [190]. Higher levels of nitric oxide in the lungs result in the activation of platelets to stimulate chronic inflammation. These conditions lead to severe damages in the endothelium of lung capillaries as well as causing microangiopathy, worsening the diabetic lung disease.

In addition to their causative and/or synergistic role in diabetes progression and complications, some of the lncRNAs have been identified as potential biomarkers for complications associated with DM. Upregulated levels of lncRNA-ARAP1-AS2, NR-033515, CDKN2B-AS1, and HOTAIR as well as downregulated levels of lncRNA-ARAP1-AS1, CASC2, GAS5, and ZEB1-AS1 have been proposed as biomarkers for DN [159]. The observations that lncRNA BANCR is downregulated while lncRNA MIAT is elevated in DM patients with DR identify them as predictive biomarkers for DR [159]. Similarly, lncRNA SRA, whose level is lower, and lncRNA ANRIL, whose level is higher in DM patients with cardiovascular complications, are being investigated as biomarkers for DM with cardiovascular ailments [159]. Thus, the diverse mechanisms of action exhibited by lncRNAs in regulating metabolic disorders, such as diabetes, attributes to its several allied disease complications as well as opens the path in exploring them as biomarkers, thus providing theoretical insights to manage DM.

## 5. Conclusions

Cellular homeostasis is maintained through a vast array of cellular signaling networks that act in concert to elicit coordinated responses in response to internal/external cues. Dysregulation of the network forms the etiological basis for most, if not all, of the diseases. Therefore, identifying the aberrant signaling nodes and the causative factors could serve as potential therapeutic targets. A greater part of our current therapeutic strategies rely on the data arising from mere 2% of the genome, which codes for the proteins. Recent studies, as discussed here, have shown that the non-coding RNAs, especially the lncRNAs, play cardinal roles in the genesis and progression of multifactorial human diseases. Each of the human diseases are complex with respect to the interconnected network of signaling pathways and their crosstalks. LncRNAs mediate the crosstalk between these diverse signaling cascades with their diverse interactome including proteins, RNAs and lipids. Aberrant expression or asynchronous signaling by lncRNAs and their interactome contribute not only to the pathogenesis and progression of diseases, but also to therapy resistance. Furthermore, similar to the SNPs (single nuclear polymorphisms) in coding regions of the genome, mutations in lncRNAs are also being unraveled to contribute to disease risks in humans. The systematic investigation of lncRNA profiles in diverse human disease types would pioneer the efforts in developing lncRNA-based disease biomarkers as well as therapeutic targets. However, extensive in vitro and in vivo experimental validations are required for definitive conclusions on the regulatory as well as biological roles of lncRNAs and the effects of targeting the critical lncRNAs for therapy. Unraveling the diverse lncRNA-based therapeutic strategies would undoubtedly shed light on the existing complexities of several targeted therapeutics that so far have been unsuccessful in suppressing the disease or even in combating the multifaceted therapeutic resistance. Considering the multitudes of genetic and epigenetic events regulated by lncRNAs, development of a personalized therapeutic approach can be refined by investigating the therapeutic targeting lncRNAs alone or in combination with other targeted therapeutic agents.

## Figures and Tables

**Table 1:** Major LncRNAs in disease pathology.

Disease	Upregulated LncRNAs	Downregulated LncRNAs
**Cardiovascular Diseases**	APF [80] BANCR [81] CHAER [82] CHAST [83] CHRF [84] CHROME [85] Giver [86] HOTTIP [87] LINC00968 [88] lnc-Ang362 [89] lnc-AK098656 [90] MIAT [91] MIRT1 [92] KCNQ1OT1 [93] UCA1 [94]	FTX [95] GAS5 [96] HOTAIR [97] lincRNA-p21 [98,99] MHRT [100] NEXN-AS1 [101] SENCR [102]
**Neurological Diseases**	BACE1-AS [103,104] C9ORF72-AS [105] EBF3-AS [106] GAS5 [107] LINC00507 [108] lincRNA-p21 [109] MALAT1 [110] MEG3 [104] MSNP1-AS [111] NEAT1 [104,112] SHANK2-AS [113] SNHG1 [114,115,116] SNHG14 [117] SOX21-AS1 [118] SYNGAP1-AS [119] TUG1 [104] XIST [104,120]	DISC1-AS [121] H19 [122] HAR1 [104] MALAT1 [123] MEG3 [124] MIAT/GOMAFU [125]
**Cancer**	ANRIL [126] BGL3 [127] CCAT1 [128] CCAT2 [129] CRNDE [130] DLEU2 [131] HULC [132] HOTAIR [133,134] linc-NMR [135] LINC00337 [136] LINC00963 [137] lnc-Hh [138] LNMAT1 [139] MALAT1 [140,141,142,143] MVIH [144] NEAT1 [145] POU3F3 [146] PVT1 [147,148] REG1CP [149] UCA1 [150,151,152,153,154]	GAS5 [155] LBCS [156] lincRNA-p21 [157,158]
**Diabetes**	ANRIL [159] ARAP1-AS2 [159] Bhmt-AS [160] CDKN2B-AS1 [159] HOTAIR [159,161] IGF2-AS [162] KCNQ1OT1 [163] LEGLTBC [164] LINC00968 [165] lnc-p3134 [166] lnc-Rpph1 [167] MALAT1 [168,169,170,171,172] MEG3 [173,174] MIAT [175] NONRATT021972 [176] NR-033515 [159] PVT1 [177] RNCR3 [178] uc.48+ [179,180]	ARAP1-AS1 [159] BANCR [159] CASC2 [159] GAS5 [181,182] H19 [183,184,185,186,187,188] HOTAIR [189] LUCAT1/SCAL1 [190] MEG3 [191,192] MIRT2 [193] SHGL [194] SNHG7 [195] SRA [159] TUG1 [196,197] ZEB1-AS1 [198]

## Data Availability

Not applicable.
